# VPT2 Calculations of Vibrational Energies of CH_3_COOC_6_H_4_COOH Done in Seconds on a Laptop Using a Machine Learned Potential

**DOI:** 10.1021/acs.jpclett.6c01186

**Published:** 2026-06-04

**Authors:** Saikiran Kotaru, Chen Qu, Apurba Nandi, Paul L. Houston, Joel M. Bowman

**Affiliations:** † Department of Chemistry and Cherry L. Emerson Center for Scientific Computation, 1371 Emory University, Atlanta, Georgia 30322, United States; ‡ Independent Researcher, Toronto, Ontario M9B0E3, Canada; ¶ Department of Chemistry and Chemical Biology, Cornell University, Ithaca, New York 14853, United States

## Abstract

The determination of quartic force fields for use in vibrational second-order perturbation (VPT2) calculations, currently available in numerous electronic structure packages, becomes very expensive as the size of the molecule increases, especially if high-level coupled-cluster theory is used. Machine-learned potentials (MLPs) for large molecules and clusters offer a viable alternative to obtaining the quartic force field (QFF). Here, we report Fortran and Python software to determine the QFF and perform VPT2 calculations of energies from the MLPs. We describe this software and then apply it to H_2_O and protonated oxalate as the test cases. The Fortran software is applied to 21-atom aspirin using a fast MLP reported by us. Despite the fact that there are 32,509 unique cubic force constants for aspirin, the computer time to calculate them using this MLP is trivial, i.e., around 1 min. The new software provides an efficient way to calculate quantum anharmonic energies, using the established VPT2 methodology, for machine learned potentials of large molecules.

## Graphical abstract

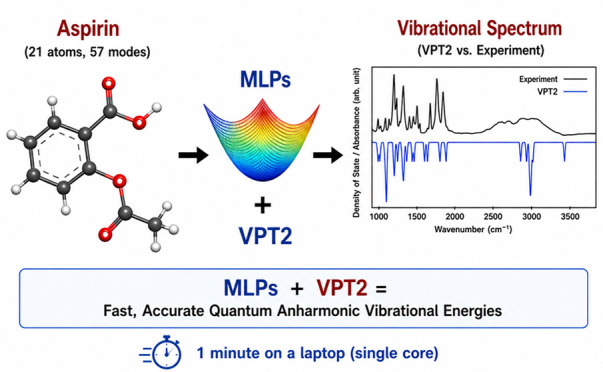

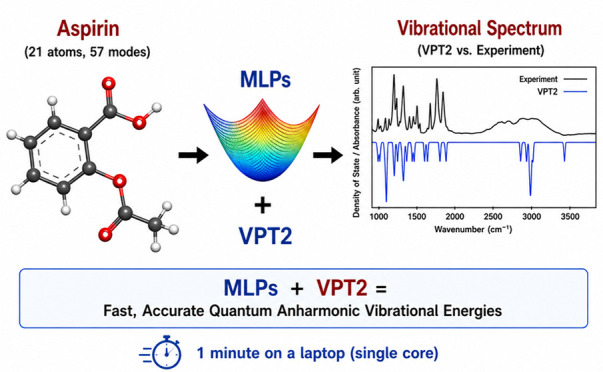

Theoretical computation of vibrational energies is essential for interpreting spectroscopic measurements and is an active area of theoretical chemistry.^1–5^ The simplest approach is the harmonic approximation in normal coordinates, which results in a quadratic separable potential in normal coordinates and therefore ignores anharmonicity and mode coupling. Vibrational second-order perturbation theory (VPT2),^6^ for which the harmonic approximation is the zero-order model, includes anharmonic effects through cubic and quartic force constants in a perturbative treatment. This is a widely used method, owing to its accuracy and also because it is very efficient computationally compared to more rigorous variational approaches. However, the direct determination of force constants in quartic force fields (QFFs), currently available in some electronic structure packages, becomes prohibitively expensive as the size of the molecule increases, especially if high-level coupled cluster energies are used.^7^ To be specific, the number of unique cubic force constants for a molecule with *N* vibrational normal modes is given by (N+23) , which scales as *N* ^3^. As noted in the abstract, this number is 32,509 for 57-mode aspirin. These force constants can be obtained using finite difference expressions using energies, gradients, or Hessians. For aspirin, using energies requires roughly 200,000 evaluations. Fewer terms are needed if gradients or Hessians are used, but these are more expensive to evaluate than energies (with the additional cost highly dependent on the level of *ab initio* theory used). In short, a direct *ab initio* calculation of the QFF for aspirin would be very computationally intensive. It should be noted, however, that efficient multilevel DFT approaches have been developed that permit VPT2 calculations with up to 50 atoms.^7,8^

Machine-learned potentials (MLPs) for large molecules or clusters, when available, offer a viable alternative to obtain QFFs (among many other uses). This is because MLPs can evaluate energies and gradients orders of magnitude faster than direct electronic structure calculations. However, one must still consider the cost to train an MLP. Although this can be substantial, because the resulting MLP can be used in many applications, this cost is “amortized” over these. Also, it should be noted that many MLPs already exist in the literature. In addition to the many MLPs targeted for a given molecule, there are also “transferable” ones that can be used for classes of molecules, such as MACE-OFF,^9^ SO3LR,^10^ and Allegro.^11^ Also we note a Δ-ML transferable many-body PIP MLP for linear alkanes.^12^ Whether targeted or transferable, MLPs can be used directly in VPT2 calculations at virtually no cost, as we show here for 21-atom aspirin. We do note that MLPs have already been used with VPT2 for “small” molecules. Meuwly and co-workers combined forces with software in Gaussian^13^ to obtain the QFFs and perform subsequent VPT2 calculations, using PhysNet potentials for a number of molecules with up to ten atoms.^14–16^

Inspired by this work, and also work by Matyus, Behler, and co-workers applying VPT2 to an MLP for formic acid dimer,^17^ we developed stand-alone software in Fortran and Python to determine the QFF using standard finite difference approaches using Hessians, gradients, or energies of MLPs. We then use Python software to perform the suite of VPT2 calculations directly using QFF. We demonstrate this software for H_2_O and protonated oxalate as tests. We then apply the software to obtain VPT2 energies for the 21-atom aspirin. To the best of our knowledge, this is the largest molecule for which VPT2 energies have been reported using an MLP.

This paper is organized as follows. We first give a brief review of QFFs, VPT2, and the two widely used methods to deal with resonances, deperturbed VPT2 (DVPT2) and generalized VPT2 (GVPT2). The new Fortran and Python software to calculate the QFF from MLPs and then to obtain the VPT2, DVPT2, and GVPT2 energies is described. Tests are presented for H_2_O and protonated oxalate, and new calculations are reported for 21-atom aspirin (57 vibrational modes).

As noted above, VPT2 is second-order perturbation theory, where the separable harmonic-oscillator Hamiltonian (with neglect of vibrational angular momentum terms) is the zeroth-order model. In this approach, the potential, *V*, is expanded as a Taylor series about a minimum in terms of the (mass-scaled) normal coordinates, denoted *q*_*i*_.^13,18,19^ Truncation at the (separable) quadratic terms is the zeroth-order model, and the cubic and quartic terms of that series enter formally as terms of order λ and λ^2^, respectively.^6^ The QFF, in principle, contains all the cubic and quartic terms; however, for practical considerations, only the semidiagonal quartic terms are retained. The cubic force constants are given by^13,18^

1 $$\phi_{ijk}=\frac{\partial^{3}V}{\partial q_{i}\partial q_{j}\partial q_{k}}$$

and the semidiagonal quartic force constants are given by

2 $$\phi_{iijj}=\frac{\partial^{4}V}{\partial q_{i}^{2}\partial q_{j}^{2}}$$

where *i*, *j*, *k* label the vibrational normal modes. As noted above, these force constants are generally obtained from direct electronic structure calculations numerically, and are by far the major source of the computational effort in VPT2 calculations.

The VPT2 fundamental frequencies (ν_*i*_) for mode *i* are given by^13,18^

3 $$\nu_{i}=\omega_{i}+2\chi_{ii}+\frac{1}{2}\underset{j\neq i}{\sum }\chi_{ij}$$

where ω_*i*_ is the harmonic vibrational frequency, and the anharmonicity constants χ_*ii*_ and χ_*ij*_ are given by

4 $$\begin{array}{l} 16\chi_{ii}=\phi_{iiii}-\underset{j}{\sum }\frac{\left(8\omega_{i}^{2}-3\omega_{j}^{2}\right)\phi_{iij}^{2}}{\omega_{j}\left(4\omega_{i}^{2}-\omega_{j}^{2}\right)} \end{array}$$

5 $$\begin{array}{l} 4\chi_{ij}=\phi_{iijj}-\underset{k}{\sum }\frac{\phi_{iik}\phi_{jjk}}{\omega_{k}}+\underset{k}{\sum }\frac{2\omega_{k}\left(\omega_{i}^{2}+\omega_{j}^{2}-\omega_{k}^{2}\right)\phi_{ijk}^{2}}{\Delta_{ijk}}+\frac{4\left(\omega_{i}^{2}+\omega_{j}^{2}\right)}{\omega_{i}\omega_{j}}\underset{\tau }{\sum }B_{\tau }^{e}{\left(\zeta_{ij}^{\tau }\right)}^{2} \end{array}$$

where $B_{\tau }^{e}$ (τ = *x*, *y*, *z*) are the equilibrium rotational constants, $\zeta_{ij}^{\tau }$ are the Coriolis coupling constants, and

6 $$\Delta_{ijk}=\left(\omega_{i}+\omega_{j}+\omega_{k}\right)\left(\omega_{i}+\omega_{j}-\omega_{k}\right)\left(\omega_{i}-\omega_{j}+\omega_{k}\right)\left(\omega_{i}-\omega_{j}-\omega_{k}\right)$$

A well-known issue with this approach is evident from eqs 4 and 5, where the energy denominators approach zero, due to the presence of resonances, e.g., when ω_*j*_ ≈ 2ω_*i*_ (Fermi type-I) or ω_*k*_ ≈ ω_*i*_ + ω_*j*_ (Fermi type-II). These near-degeneracies can lead to large errors in the computed vibrational energies. Methods to deal with resonances have been made and applied for at least 30 years and these are known as “deperturbed VPT2” (DVPT2) and “generalized VPT2” (GVPT2).^8,13,19–23^ In DVPT2, resonant contributions are removed, while GVPT2 subsequently treats the interacting resonant states by small, e.g., 2 × 2 or 3 × 3 “configuration interaction” calculations. The extensive developments of VPT2 and extensions beyond energies to numerous properties^8^ have made VPT2 a powerful and popular approach for anharmonic vibrational analysis.

As noted above, we have written stand-alone software in Fortran and Python to create a two-stage workflow to perform VPT2 calculations using an MLP. Details of this workflow are as follows. In step one the QFF is calculated. The mass-scaled normal modes are obtained as usual by diagonalizing the mass-scaled Hessian. In our Python software the normal modes are obtained analytically from the Hessian, using the ASE calculator. In our Fortran software, they are obtained using finite differences of analytical gradients in mass-scaled Cartesian coordinates. These normal modes are used to obtain the cubic and semidiagonal force constants, using finite differences approximations in both the Fortran and Python software. In the Fortran implementation, the cubic and semidiagonal force constants are obtained using finite differences of numerical Hessians, obtained using analytical gradients. In the Python software, the cubic and semidiagonal quartic force constants are obtained from finite differences using the analytical Hessian, which is typically available in many Python-based MLPs, including PhysNet which is used here for the protonated oxalate anion test. Note, we ran numerous calculations varying the finite-difference step sizes and finite difference expressions for the test cases reported here to determine converged values used in our software. (These can be changed by the user.) The second step uses Python software to read the QFF and to perform VPT2, DVPT2 and GVPT2 calculations of vibrational energies. A schematic of the workflow of the Python software is given in Figure 1. We note that this Python software adapts sections of the Python software PyVPT2,^24^ and uses the “Martin” test^20^ for the treatment of Fermi resonances. We use a resonance threshold energy of 100 cm^–1^ and a corresponding resonance coupling greater than 1.0 cm^–1^. These quantities are explained in detail in the 1995 paper.^20^ Note that PyVPT2 makes direct calls to electronic energy software, e.g., PSI4, to obtain the QFF. This is distinct from our Python software, which calculates the QFF from an MLP.

**Figure 1:**
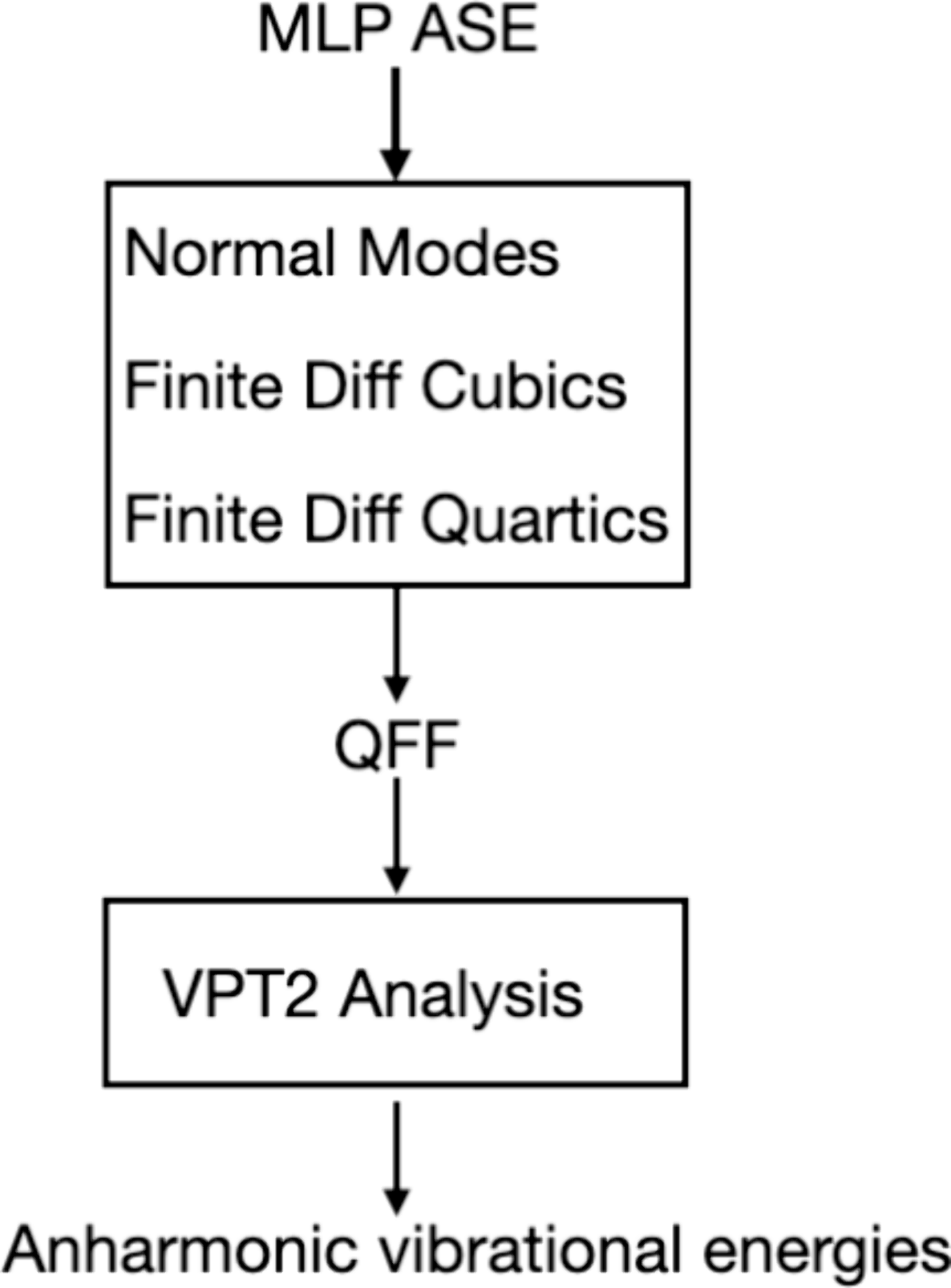
Schematic of Python software workflow.

The software is applied below for two tests: one for H_2_O and the other for protonated oxalate anion. Then, we apply the software to obtain VPT2, DVPT2, and GVPT2 energies for the 57 fundamentals of the 21-atom aspirin using our MLP.^25^ Comparisons with experiments are given.

Tests for H_2_O were done using the spectroscopically accurate potential of Partridge and Schwenke.^26^ This is a simple example, as there are no resonances for the fundamentals. However, the effects of Coriolis coupling are significant owing to the large A-rotation constant. The QFF was obtained using energies only and high-order finite difference approximations. VSCF/VCI calculations using MULTIMODE^27^ were also performed and the results of the VPT2 and those calculation with and without Coriolis coupling are given in Table 1. As seen there is very good agreement with the VSCF/VCI results (denoted “VCI” in the table).

**Table 1:** VPT2 and VCI Fundamental Energies (cm^–1^) Obtained with and without Coriolis Coupling (CC) for Water

Vibration Mode	VPT2 no CC	VPT2 with CC	VCI no CC	VCI with CC
bend	1580.8	1594.2	1582.1	1594.0
symm stretch	3654.4	3654.4	3656.9	3656.0
asymm stretch	3739.7	3753.0	3742.8	3755.0

Next, consider the 7-atom protonated oxalate anion where resonances play a role for some fundamentals; however, where Coriolis effects are very small and thus ignored, owing to the small rotation constants. We first present results using a PhysNet neural network potential reported in ref. 16. This potential was initially trained with MP2/aug-cc-pVTZ energies and forces at 22,100 configurations and then transfer-learned with CCSD­(T)/aug-cc-pVTZ energies and gradients at a subset of 2688 configurations. This potential is written in Python, with analytical Hessian available via autodifferentiation. The QFF was obtained using finite differences applied to the Hessian.

In Table 2, fundamentals from VPT2, DVPT2, and GVPT2 calculations using our Python software are compared with those from ref 16 which used Gaussian, with the same PhysNet MLP. As seen, there are some substantial differences between VPT2 and DVPT2 and GVPT2 results. This is due to strong effects of resonances which are ignored in VPT2. Fundamentals using DVPT2 are in good agreement with the GVPT2 ones, but with 10–20 cm^–1^ discrepancy in certain modes. Finally our GVPT2 are within 5 cm^–1^ or less of the GVPT2 results of ref 16.

**Table 2:** Fundamental Energies (cm^–1^) Computed Using VPT2, DVPT2 and GVPT2 for Protonated Oxalate Using PhysNet Potential

Number	VPT2	DVPT2	GVPT2	GVPT2^a^
1	102.49	99.45	99.45	98.74
2	291.55	291.45	291.46	288.14
3	416.03	416.03	416.03	417.97
4	479.55	478.40	478.40	474.20
5	538.71	554.85	538.91	535.99
6	690.99	690.88	690.89	691.15
7	813.97	813.92	813.93	813.99
8	840.47	840.40	840.41	838.94
9	938.92	938.66	938.66	936.56
10	1102.21	1096.00	1091.39	1089.69
11	1277.33	1307.08	1302.46	1301.92
12	951.343	1398.29	1383.62	1381.40
13	1691.66	1691.37	1691.37	1695.42
14	1837.74	1771.41	1763.27	1763.54
15	2812.92	2765.26	2762.10	2767.01

a Reference 16.

Given the good test results for H_2_O and protonated oxalate, we applied our software to aspirin. To obtain the QFF for aspirin, depicted in Figure 2, we used our MLP written in Fortran.^25^ This MLP used permutationally invariant polynomial (PIP) regression to precisely fit energies and gradients from the rMD17 database for aspirin.^28,29^ In brief, the potential surface is given by

7 $$V\left(y\right)=\overset{n_{p}}{\underset{i=1}{\sum }}c_{i}p_{i}\left(y\right)$$

where *c*_*i*_ are linear coefficients, *p*_*i*_ are PIPs, *n*_*p*_ is the total number of polynomials (and linear coefficients *c*_*i*_) for a given maximum polynomial order, and ***y*** are transformed, Morse-like variables of internuclear distances *r*_*ij*_ between atoms *i* and *j*, i.e., *y*_*ij*_=exp­(−*r*_*ij*_/*a*).^30^ The coefficients *c*_*i*_ were determined using a standard linear least-squares approach. For the interested reader, details of this approach,^30–33^ as well as comparisons with other machine-learned approaches have been given.^25,34,35^

**Figure 2:**
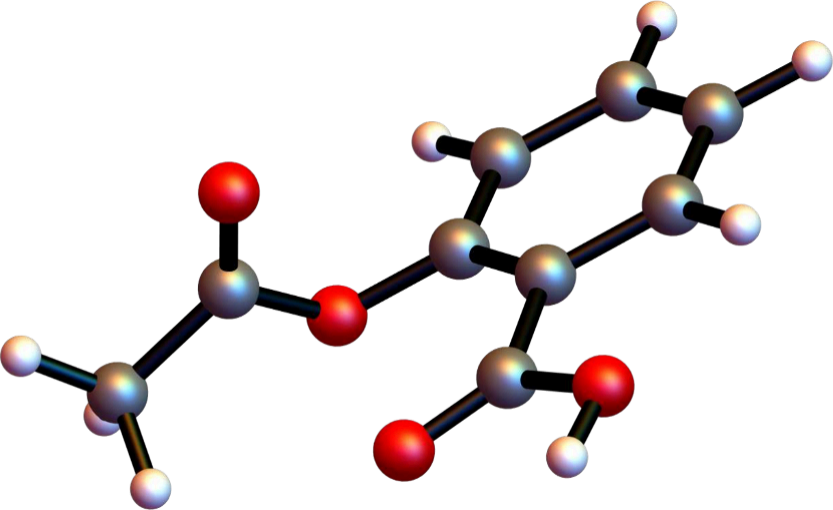
Equilibrium configuration of aspirin: white = H, red = O, gray = C.

The data set of energies and gradients for aspirin used for training were downloaded from the rMD17 data set.^28,29^ These were obtained using NVT direct-dynamics calculations at 500 K, using PBE + TS-vdW^36^ electronic structure method with the def2-SVP basis. The potential energy distribution for these geometries extends to almost 14,000 cm^–1^. We reported several PIP PESs,^25^ and the most precise fit is used here. This one contains 49,977 linear coefficients and is fit to 80,000 energies and 5,000 gradients for a total data size of 395,000. The precision of the PES fit as compared to DFT values is given by eRMSE = 27 cm^–1^ and gRMSE = 53 cm^–1^/bohr and by *R* ^2^ correlation coefficients of 0.999682 for energies and 0.999856 for gradients. Further details are given in ref 25.

Before presenting the results of the VPT2 analysis for aspirin, we note how easily and fast it is to use VPT2 theory. As noted in the title, it took less than 1 min to calculate the QFF and perform the VPT2 analysis on a desktop using a single core of Intel i7-12700K CPU.

Energies of the 57 fundamentals from the GVPT2 and harmonic analysis are shown graphically in Figure 3. The numerical results and assignment of the higher-frequency fundamentals are given in the Supporting Information (SI). The VPT2 energies are all below the harmonic ones. The difference ranges from 1.9 cm^–1^ for the lowest harmonic frequency of 34 cm^–1^, to 245 cm^–1^ for the highest harmonic frequency of 3652 cm^–1^. And we believe the accuracy of the VPT2 energies is now mostly limited by the quality of the electronic structure method used to obtain the data for the MLP.

**Figure 3:**
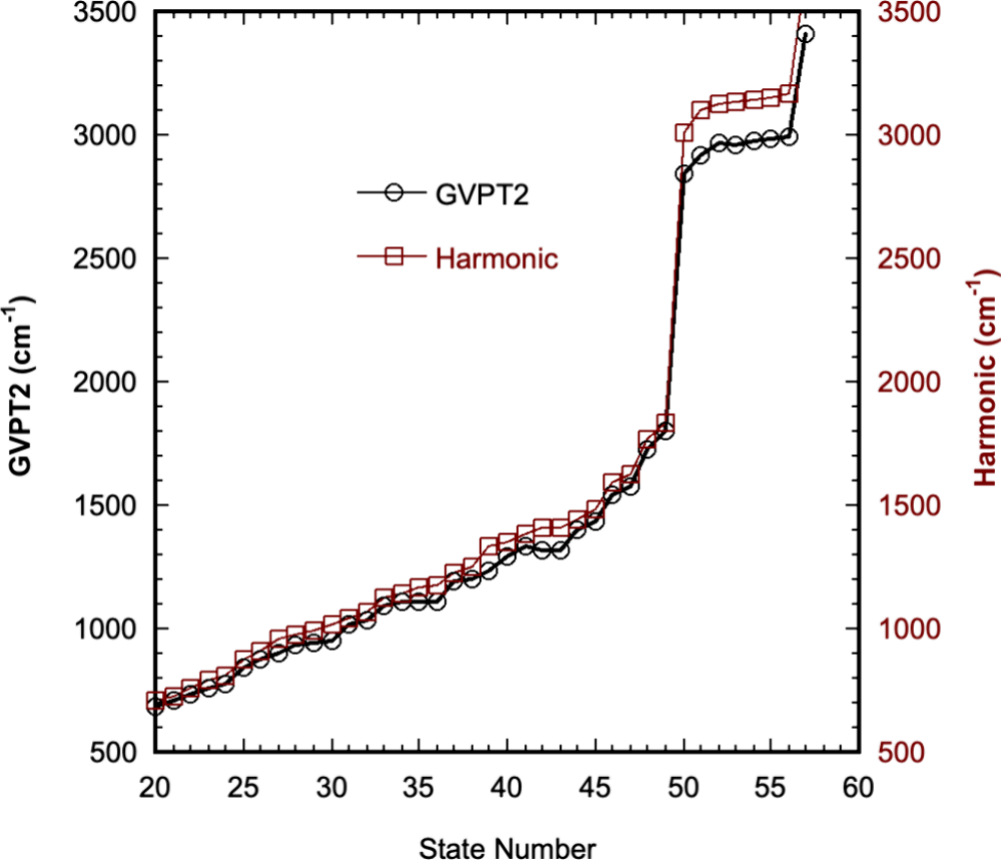
VPT2 and harmonic fundamental energies of aspirin.

The IR spectrum of aspirin in condensed phase has been reported.^37^ This spectrum is shown in Figure 4 along with the vibrational power spectrum from the GVPT2 and harmonic energies of the fundamentals. As seen, the GVPT2 spectrum aligns significantly better than the harmonic one with experiment, especially in the region of the broad higher frequency band between ca. 2800 and 3200 cm^–1^. This is the region where GVPT2 fundamental energies are significantly below the harmonic ones by ca. 150–200 cm^–1^. The fundamentals in this band (see Table S1 in the Supporting Information (SI)) are seven CH-stretches from the methyl group and the ring. Also, the gap in the IR spectrum between 2000 and 2500 cm^–1^ is reasonably reproduced by the GVPT2 power spectrum. An analysis of the origin of the very broad diffuse band centered at around 2900 cm^–1^ is beyond the scope of this paper, but clearly worth investigation.

**Figure 4:**
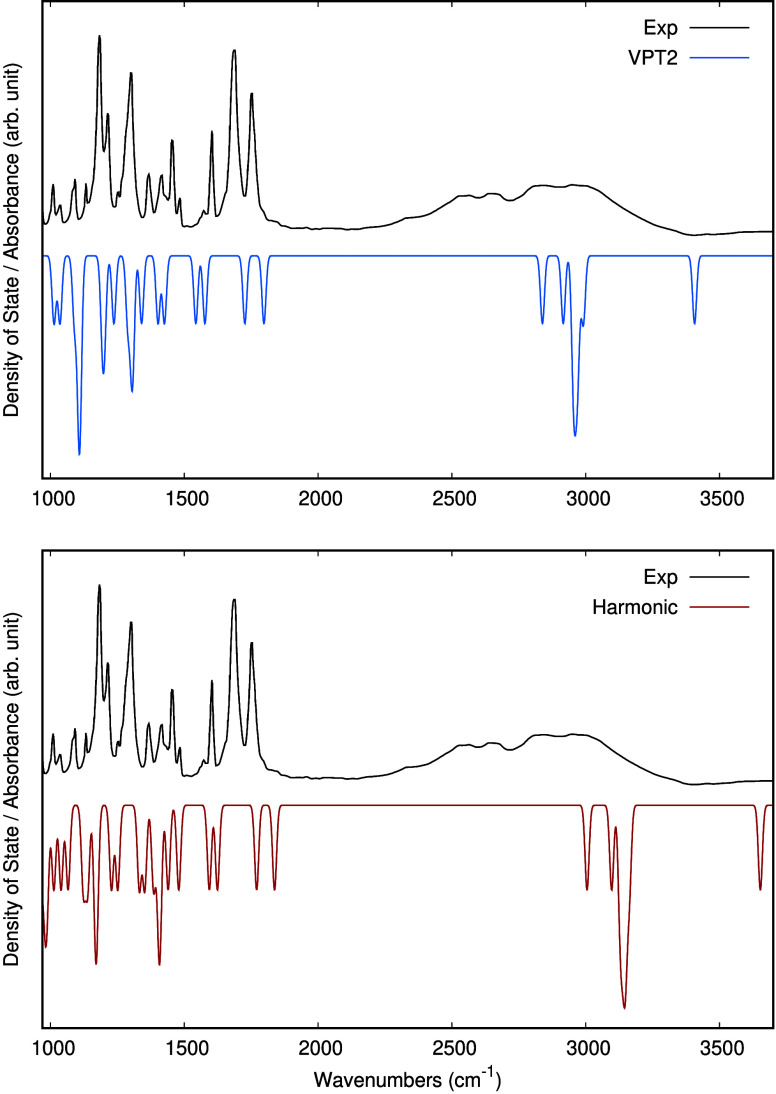
Experimental aspirin IR spectrum^37^ (black) and smooth density of states of VPT2 (blue) and Harmonic (red) of fundamental energies.

The present results are a significant step in breaking the barrier to efficiently (indeed almost trivially) apply VPT2 to large and even very large (ca. 100 atoms or more) molecules by using the recent advances in developing MLPs for such molecules. However, there are important caveats to be noted with this approach. In addition to the comments made about the cost to train MLPs, mentioned above, we note a second caveat: the accuracy of an MLP. There are two aspects here: one is the accuracy of the underlying electronic structure method used to obtain the data for the fit, and the second is the precision error of the fit. The second aspect is always addressed; however, results can vary significantly depending on the ML method used.

To examine this sensitivity, we return to protonated oxalate for which a recent PIP PES, written in Fortran, was reported.^38^ This PES was trained on the same data sets as the PhysNet potential; details of fit are given in the SI of ref 38 for the interested reader. We used this PES to obtain a QFF, using our Fortran software, with finite differences on analytical gradients as well as just energies. The resulting GVPT2 energies for the PIP PES are given in Table 3, along with those using the PhysNet potential, given in Table 2. As seen, with the exception of mode 15, results from PhysNet and PIP PES using gradients agree to within 0–3 cm^–1^. The mean absolute difference with GVPT2 energies using the PhysNet PES in the first column are also given. As seen, they are uniformly small, albeit largest for the QFF using finite difference on PIP energies, where the largest difference is 10 cm^–1^ for the lowest frequency mode. Overall, the very good agreement between the PhysNet and PIP PESs is gratifying, as these PES are based on very different regression methods (see refs 16 and 38 ) but produce high fitting precision. Specifically, for CCSD­(T) electronic energies up to 1000 cm^–1^ above the minimum, the RMSEs are 0.39 cm^–1^ for the PhysNet PES and 0.49 cm^–1^ for the PIP PES. Returning to the somewhat larger differences for mode 15, the OH-stretch, it has been noted that this mode is very strongly coupled to other modes.^16,38,39^ In fact, VPT2 including GVPT2 breaks down for this mode, as the coupling is so strong such that this band is completely fractionated. So, it is likely that the 5–10 cm^–1^ differences are due to this strong coupling. A detailed examination of the QFFs might be very interesting for a future study.

**Table 3:** Comparison of GVPT2 Fundamental Energies of Protonated Oxalate from Indicated Sources and Mean Absolute Difference (MAD) with Respect to Column 1 Energies^a^

	PhysNet^a^	PhysNet/G^b^	PIP_PES_g^c^	PIP_PES_e^d^
1	99.5	98.7	99.9	110.0
2	291.5	288.1	289.8	285.4
3	416.0	418.0	413.8	420.6
4	478.4	474.2	478.3	473.2
5	538.9	536.0	539.3	538.4
6	690.9	691.2	691.1	692.9
7	813.9	814.0	816.6	818.9
8	840.4	838.9	838.1	837.4
9	938.7	936.8	939.1	940.3
10	1091.4	1089.7	1089.4	1085.1
11	1302.5	1301.9	1304.3	1317.7
12	1383.6	1381.4	1383.2	1381.6
13	1691.4	1695.4	1691.7	1692.6
14	1763.3	1763.5	1763.5	1762.6
15	2762.1	2767.0	2756.2	2756.8
				
MAD		2.1	1.4	4.6

a Energies are in cm^–1^.

b PhysNet PES using our software:

c PhysNet PES using Gaussian software as described in ref 16.

d PIP PES using our software and finite difference of analytical gradients.

e PIP PES is our software and finite difference of energies.

The tentative conclusion from this study is that MLPs trained precisely on a data set including high energies may produce quantitatively useful QFFs; however, for states with strong resonance interactions, the results probably should be viewed with some caution. As noted above for the aspirin data set, the lower energies provided in the rMD17 data set may actually be advantageous for QFF calculations. (This observation is an updated and positive observation about this data set, which we earlier critiqued as being too limited for some uses.^40^)

We conclude this section by stressing that the new software can also be used directly on so-called universal force fields, such as MACE,^9^ SO3LR,^10^ and Allegro,^11^ with the caveats about accuracy and precision kept in mind. And because the calculation of the QFF and subsequent VPT2 analysis is very fast given an MLP, it is clear that the speed of evaluation of energies and gradients from any MLP is essentially irrelevant. Also, beyond analysis at the minimum for anharmonic energies, we note that VPT2 theory can also be used at saddle points to obtain semiclassical tunneling corrections to standard transition state theory^41^ and an interesting extension to tunneling splittings.^42^ This can also be done now for MLPs of large molecules, with the usual caveat about the accuracy of saddle points on those potentials. Also, the results for aspirin may also serve as benchmarks for approximate VPT2 approaches for larger molecules, for example, approaches using a reduced number of modes, spectator modes, etc. as discussed in refs 7 and 43 , or a fragmented local monomer approximation.^44^ Looking ahead, we plan to extend the software reported here specifically for MLPs to obtain IR intensities (and in general other properties beyond energies). This is a straightforward task, as the expressions for doing so have been well established in the literature; see refs 23, 45 for the current status. With respect to the IR spectrum there are several options. One simple, and often accurate approach, is to simply shift the double-harmonic spectrum, which is efficiently obtained in many electronic structure codes, by the VPT2 energy shifts. Tests of the intensities from the double harmonic approximation vs anharmonic dipole and VPT2 ones show good accuracy of the former.^23^ This spectrum is restricted, however, to just the fundamentals. We have done this in work being written up,^46^ where the IR spectrum of *trans*-*N*-methylacetamide was obtained using MACE-OFF^9^ and our PIP MLP,^47^ for the VPT2 calculation of energies and CCSD­(T)-based double-harmonic intensities. Agreement with the gas-phase IR spectrum is gratifyingly good. The second option is to make use of nonlinear dipole moments that are available in some MLPs, for example, PhysNet,^48^ or calculated as separate machine learning representations, e.g., see ref 33 for a PIP approach that has been used to obtain general dipole moments of molecules, clusters and even water. Expressions and software to use nonlinear and multimode dipole with VPT2 to describe overtones and combination bands, have been developed and applied, in for example refs 23 and 45 . We conclude these comments about future developments by noting significant earlier and related work in 1985 by Harding and Ermler. They reported a Fortran code “SURVIB”^49^ in which a very precise fit is made using to a grid of electronic energies at configurations very close to the minimum. The fit is then used in a normal-mode analysis, and elements of a QFF are obtained using finite-difference expressions for cubic and quartic force constants. These are then used in standard VPT2 calculations. The code was demonstrated for formaldehyde using a low level of *ab initio* theory, by current standards. Forty years later we are in the era of MLPs for molecules much larger than formaldehyde, but the software we report here is “ancestrally” related to this early, pioneering work.

To summarize, we reported software to obtain a quartic force field from general machine-learned potentials and then used it in VPT2 calculations of vibrational energies. The software was tested on H_2_O and the protonated oxalate anion and was then applied to 21-atom aspirin using a previous PIP potential. With this potential in hand the calculation of the QFF and VPT2 energies took roughly 1 min of CPU time on a desktop computer. Differences with the harmonic energies for 57 fundamentals range from 2 to 245 cm^–1^. Comparison with the only available IR spectrum shows major improvement of the GVPT2 energies compared to the harmonic ones.

## Supplementary Material



## Data Availability

The new software, written in Fortran and Python, is available on request to the authors and will eventually be made available on Github. The QFF for aspirin is also available on request from the authors.

## Notes

Table S1 as referenced in the text (PDF)
