# Supplementary material for: VPT2 Calculations of Vibrational Energies of CH3COOC6H4COOH Done in Seconds on a Laptop Using a Machine Learned Potential
Source: J Phys Chem Lett. 2026 Jun 4;17(24):6580–6. doi: 10.1021/acs.jpclett.6c01186 (PMC13288632; doi:10.1021/acs.jpclett.6c01186)
Supplement: Supplementary file 1 [file jz6c01186_si_001.pdf]

Supporting Information VPT2 Calculations of Vibrational Energies of $CH_3COOC_6H_4COOH$ Done in Seconds on a Laptop Using a Machine Learned Potential

Saikiran Kotaru ,[†] Chen Qu,[*,‡] Paul L. Houston,[*,¶] and Joel M. Bowman[*,†]

†*Department of Chemistry and Cherry L. Emerson Center for Scientific Computation, Emory University, Atlanta, Georgia 30322, U.S.A.*

‡*Independent Researcher, Toronto, Ontario M9B0E3, Canada*

¶*Department of Chemistry and Chemical Biology, Cornell University, Ithaca, New York 14853, U.S.A.*

E-mail: szquchen@gmail.com; plh2@cornell.edu; jmbowma@emory.edu

Table 1: Fundamental frequencies (cm^{-1}) of aspirin computed using different resonance treatments.

Number	HO	VPT2	DVPT2	GVPT2
1	34.670	32.760	32.760	32.760
2	85.070	80.810	80.790	80.790
3	90.450	86.550	86.540	86.540
4	100.66	95.930	95.920	95.920
5	128.26	110.22	110.16	110.16
6	137.92	134.74	134.74	134.74
7	226.28	222.07	222.07	222.07
8	262.93	255.62	255.61	255.61
9	305.71	298.68	298.67	298.67
10	354.51	343.59	343.58	343.58
11	414.27	405.85	405.83	405.83
12	426.10	411.89	411.88	411.88
13	502.88	486.64	486.62	486.62
14	519.09	496.96	496.95	496.95
15	545.07	531.15	531.14	531.14
16	563.19	533.72	533.72	533.72
17	595.60	569.65	569.15	569.15
18	628.56	609.06	609.06	609.06
19	656.87	634.60	637.06	637.13
20	706.93	679.43	679.18	679.18
21	727.60	704.73	704.72	704.72
22	759.98	732.72	732.71	732.71
23	793.07	756.52	756.36	756.36
24	812.39	773.55	786.03	787.59
25	877.11	839.88	839.87	839.87
26	907.14	874.65	874.64	874.64
27	961.58	898.36	898.36	898.36
28	978.50	929.71	927.93	927.77
29	987.71	938.76	938.76	938.76
30	1012.8	946.89	946.89	946.89
31	1039.7	1013.5	1013.5	1013.5
32	1065.9	1036.2	1035.4	1035.6
33	1123.0	1090.0	1090.0	1090.0

Table 1 continued.

Number	HO	VPT2	DVPT2	GVPT2	Approximate Description
34	1139.0	1106.5	1106.5	1106.5	
35	1167.9	1107.9	1107.9	1107.7	
36	1173.3	1109.3	1111.6	1111.5	
37	1228.1	1194.0	1194.0	1194.0	
38	1251.5	1200.0	1202.3	1201.9	
39	1332.2	1237.1	1237.1	1237.1	
40	1352.2	1294.6	1287.8	1289.1	
41	1385.5	1332.5	1338.5	1340.6	
42	1404.7	1313.5	1303.8	1303.8	
43	1410.2	1314.2	1308.3	1308.2	
44	1439.7	1402.0	1402.0	1402.0	
45	1479.4	1429.5	1426.5	1425.8	
46	1593.7	1543.2	1543.2	1543.2	
47	1623.8	1577.4	1577.2	1577.2	
48	1770.5	1725.1	1726.4	1726.4	C=O str. (carboxy)
49	1837.3	1797.6	1797.6	1797.6	C=O str.(ester)
50	3004.8	2838.1	2838.1	2838.1	CH str. (CH_3)
51	3096.8	2915.8	2915.8	2915.8	CH str. (CH_3)
52	3127.4	2965.3	2960.2	2953.5	CH str.(ring)
53	3135.0	2961.4	2953.7	2956.2	CH str. (CH_3)
54	3145.2	2971.2	2971.2	2971.2	CH str.(ring)
55	3149.2	2984.6	2974.5	2965.5	CH str.(ring)
56	3162.7	2990.9	2990.9	2990.9	CH str.(ring)
57	3652.2	3408.0	3406.5	3406.5	OH str.
